# Retraction of: Esophageal carcinoma cell-excreted exosomal uc.189 promotes lymphatic metastasis

**DOI:** 10.18632/aging.206351

**Published:** 2025-12-31

**Authors:** Zhiyan Ding, Yun Yan, Yu Lian Guo, Chenghai Wang

**Affiliations:** 1 Department of Pathology, The Affiliated Hospital of Yangzhou University, Yangzhou University, Yangzhou 225009, PR China; 2 Institute of Translational Medicine, Medical College, Yangzhou University, Yangzhou 225001, PR China; 3 Jiangsu Key Laboratory of Integrated Traditional Chinese and Western Medicine for Prevention and Treatment of Senile Diseases, Yangzhou University, Yangzhou 225001, PR China

**Keywords:** exosomal uc.189, HLECs, lymphangiogenesis, metastasis esophageal carcinoma

**This article has been retracted:** The journal’s investigation was initiated following a retraction request from the corresponding author Chenghai Wang. The stated reason for this request was “misquoted a fund (Chinese National Nature Science Foundation, No. 81471547).” Our investigation by the Scientific Integrity office identified further concerns:

- Image forensics analysis found image manipulations, including blurring and splicing, in the Western blot (WB) images presented in Figure 6D.
- The WB images used in Figure 4D were reused from Figure 6D of a paper published in Aging in 2020 [1], which shares one co-author, Chenghai Wang.

The Scientific Integrity office attempted to contact the authors regarding these concerns but did not receive a reply. The editorial decision was made to retract the article, and the authors were notified of this decision.
